# Early indicators of survival following exposure to mustard gas: Protective role of 25(OH)D

**DOI:** 10.1016/j.toxlet.2016.02.013

**Published:** 2016-03-02

**Authors:** Lopa. M. Das, Amy. M. Binko, Zachary. P. Traylor, Lori. R. Duesler, Scott. M. Dynda, Sara Debanne, Kurt. Q. Lu

**Affiliations:** a Department of Dermatology, School of Medicine, Case Western Reserve University, 10900 Euclid Avenue, Cleveland, OH 44106, United States; b Department of Epidemiology and Biostatistics, School of Medicine, Case Western Reserve University, 10900 Euclid Avenue, Cleveland, OH 44106, United States

**Keywords:** Sulfur mustard, Inflammation, Mortality model, Intervention, 25(OH)D

## Abstract

The use of sulfur mustard (SM) as a chemical weapon for warfare has once again assumed center stage, endangering civilian and the military safety. SM causes rapid local skin vesication and late-onset systemic toxicity. Most studies on SM rely on obtaining tissue and blood for characterizing burn pathogenesis and assessment of systemic pathology, respectively. However the present study focuses on developing a non-invasive method to predict mortality from high dose skin SM exposure. We demonstrate that exposure to SM leads to a dose dependent increase in wound area size on the dorsal surface of mice that is accompanied by a progressive loss in body weight loss, blood cytopenia, bone marrow destruction, and death. Thus our model utilizes local skin destruction and systemic outcome measures as variables to predict mortality in a novel skin-based model of tissue injury. Based on our recent work using vitamin D (25(OH)D) as an intervention to treat toxicity from SM-related compounds, we explored the use of 25 (OH)D in mitigating the toxic effects of SM. Here we show that 25(OH)D offers protection against SM and is the first known demonstration of an intervention that prevents SM-induced mortality. Furthermore, 25 (OH)D represents a safe, novel, and readily translatable potential countermeasure following mass toxic exposure.

## 1. Introduction

Sulfur mustard (SM) has been used as a chemical weapon in World War I and during inter-war periods causing life-threatening injury for which there is currently no specific treatment. Indeed the recent past has once again witnessed the horrors of SM re-emerging as an arsenal for combat so the pressure is on, more than ever, to intensify the search for effective intervention. In this study we use SM as the inducer of chemical injury which possesses a range of toxicities with exposure to varying intensities. Low dose cutaneous exposure to SM results in erythema and burning (Borak and Sidell, 1992; Requena et al., 1988), while higher doses are associated with full thickness necrosis, skin vesication and disruption of the dermal-epidermal junction (Sidell et al., 1997). Massive exposures lead to systemic toxicity and death. Despite these studies, the threshold of dose exposure that would generate a reproducible local and systemic tissue injury is not well established. We have recently shown in a nitrogen mustard (NM) induced skin wound model that 5 ng 25(OH)D, when administered intraperitoneally at 1 h following exposure, accelerated wound healing and rescued mice from mortality in an inducible nitric oxide synthese (iNOS)-dependent manner. A single dose of 25(OH)D accelerated skin wound healing, recovery from NM-mediated bone marrow disruption and pancytopenia and prevented a precipitous drop in body weight and subsequent death (Au et al., 2015). Since NM share similar vesicant and toxicity profiles with SM, various studies have utilized NM to serve as the less aggressive surrogate toxicant of SM (Goswami et al., 2015; Malaviya et al., 2012). Our model demonstrates that early effects of SM exposure causes extensive erosion of the epidermal layer of skin, inflammation and full thickness necrosis while late effects are associated with massive disruption of hematopoiesis leading to death as the end point. To date there have no effective counter-measures that offer protection from SM mediated death. Given our LD70 dose of SM by day 6 following exposure, we explored the use of one dose of 25(OH)D and demonstrate significant protection from lethality. Most studies in experimental animal models evaluate tissue damage from SM exposure from invasive skin biopsies, blood and bone marrow aspirates however the present model focuses on developing a non-invasive method to predict mortality from high dose cutaneous SM exposure.

## 2. Materials and methods

### 2.1. Mice

Pathogen-free, six week old female C57/BL6J mice were purchased from Jackson Laboratories (Bar Harbor, Maine). Animals were quarantined according to Battelle standard operating procedure (SOP). All animals receive standard laboratory diet. Anesthetic calculations were based on weights obtained during quarantine. An anesthesia cocktail of a 10 ml/kg mixture of ketamine hydrochloride (Fort Dodge, 100 mg/ml) and xylazine hydrochloride (Lloyd, 20 mg/ml) was administered by intraperitoneal (IP) injection. Hair on the dorsal back of mice was removed by clippers and depilation as previously described (Au et al. JID. 2015). Mice were allowed to rest for 72 h before initiating SM exposure experiments.

### 2.2. Sulfur mustard

SM was supplied by U.S. Army Edgewood Chemical Biological Center (Aberdeen Proving Ground, MD). The SM purity value was >96 percent as determined by gas chromatography. As part of the dose finding study, SM dissolved in DMSO [Hyclone; Logan, UT], was applied at 8 different concentrations (4.8, 8.2, 14, 23.6, 40, 68, 89 and 116 mg/kg) over an area approximately 8 mm in diameter on the dorsal surface of each mouse. To determine a stable LD70 model, doses of 50, 55 and 60 mg/kg were applied on mice. Control mice were applied DMSO only.

### 2.3. Vitamin D

25(OH)D (Sigma-Aldrich) was reconstituted in ethanol and further diluted in mineral oil Sigma-Aldrich Chemical Company, Inc. (St. Louis, MO), for use. 5 ng of 25(OH)D was injected intraperitoneally using a 27 gauge needle (Beckton Dickinson Bioscience; Franklin Lakes, NJ) 1 h following SM exposure. Untreated mice receiving SM only were injected with mineral oil.

### 2.4. Wound area size and total body weight measurements

Wounds were measured with a digital caliper daily for 5 days following SM exposure. All mice in the study were weighed daily till day 5 or till drop in body weight met euthanasia criteria.

### 2.5. Blood smear

A drop of blood was collected from SM exposed and control mice and transferred onto a glass slide by tail snip, smeared using a microscope slide (Fisher Scientific), fixed in 100% methanol (Fisher Scientific), and stained with Wright-Giemsa to observe cell types and enumerate red blood cells per high power field (HPF).

### 2.6. Complete blood count (CBC)

Blood was collected from mice undergoing sacrifice on day 5 and analyzed for complete blood count using a hematology analyzer at Battelle R&D facility (Columbus, OH)

### 2.7. H&E staining

Sternums and skin of euthanized mice were removed and fixed overnight in 10% formalin diluted in PBS (Fisher Scientific). Sternums were embedded in paraffin, sectioned (8 µm thickness), stained with H&E and observed under the microscope to evaluate SM-induced cellular depletion.

### 2.8. Immunofluorescence staining

Freshly harvested skin and sternum designated for immunofluorescence studies were OCT-embedded and analyzed as previously described (Au et al. 2015). Primary antibodies were diluted in 10% goat serum: anti-mouse F4/80 antigen Alexa-488; rat IgG2a isotype—Alexa 488 (eBioscience, SanDiego, CA, 1:100); rabbit-anti-mouse iNOS (Upstate Temecula, CA); isotype rabbit IgG (R&D Systems Minneapolis, MN). Secondary antibodies: goat anti-rabbit Alexa Fluor 647 conjugated (1:2000 in 1× PBS) (Life Technologies Grand Island, NY). Labeled sections were imaged using a Ultra-VIEW VoX spinning disk confocal microscope (Leica DMI6000B).

### 2.9. Bone marrow cell isolation

Tibiae and femurs of mice were isolated to harvest bone marrow cells as described before (Horak et al., 1983). Viable cells were enumerated in SM-exposed mice and compared with controls.

### 2.10. Statistical analysis

A 2 sided unpaired t-test was used to compare mean values. The Kaplan-Meier method was used to plot the survival distributions of all groups (SM and SM + 25(OH)D) which were then compared using the log-rank test. A Cox regression analysis was used to assess mortality hazard as a function of lesion size and body weight loss. Data are presented as mean ± s.e.m, and p values ≤0.05 were considered significant.

## 3. Results

### 3.1. SM-induced skin injury is exacerbated by infiltration of inflammatory macrophages

**Fig. 1:**
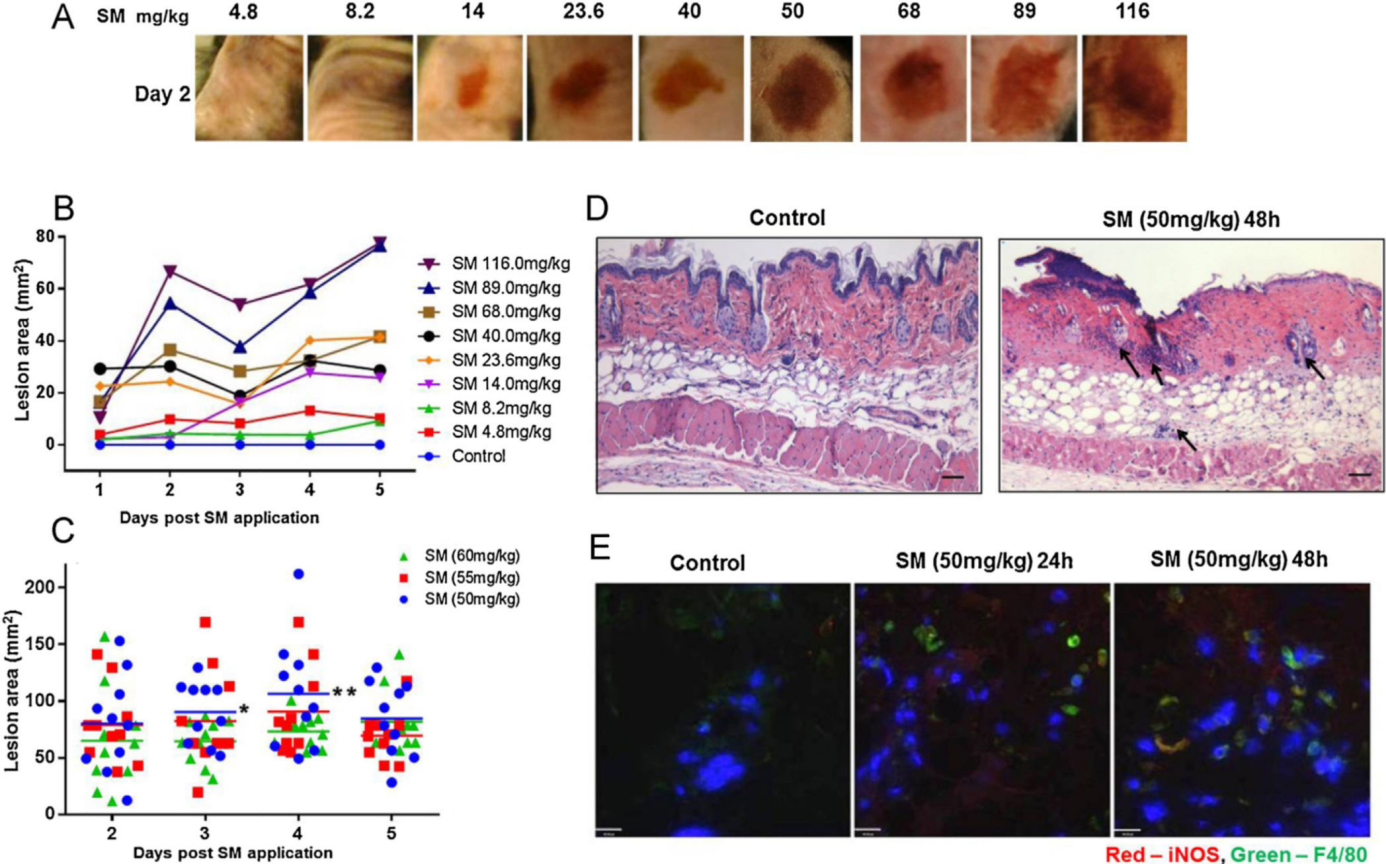
SM-induced skin injury is exacerbated by infiltration of inflammatory macrophages. Presented in a dose dependent manner are A) representative images of skin wounds 48 h post SM exposure, **B**) SM-induced lesion area measured over a 5 day period, n = 4 per group. C) The threshold dose of SM was refined to ensure an LD_90_ model. Lesion area measured in mice treated with selective doses of 50, 55 and 60 mg/kg SM, n = 10, *p = 0.025 comparing 50 mg/kg:60 mg/kg on day 3, **p = 0.05 comparing 50 mg/kg:60 mg/kg on day 4. Exposure to 50 mg/kg rendered a stable LD_70_ model, therefore subsequent experiments utilized 50 mg/kg SM as dose of exposure. These included **D**) H&E skin sections from excised biopsies obtained after 48 h post SM exposure. Arrows indicate cellular infiltration. **E**) Confocal images of epidermis/dermis (upper panel) of control vs. SM-exposed mouse skin colocalizing F4/80^+^ macrophages and iNOS^+^ cells. Scale bar = 100uM.

We developed a SM skin exposure mouse model in which dorsally shaved mice received 8 different concentrations of SM topically applied (in DMSO) onto a 50 mm^2^ circular area (8 mm diameter). The challenge dose range (4.8–116 mg/kg SM) was selected in order to reduce confidence limits around LD_90_ and obtain a statistically significant probit slope. Skin lesions were evident in all SM challenged groups with representative images shown in Fig. 1A.

Wounds progressed with visible erythema associated with hemorrhagic crust that remained enlarged resulting in full thickness necrosis and a dose dependent increase in wound area size from 4 to 70 mm^2^ (Fig. 1B). We selected 50 mg/kg SM as the working dose as this produced statistically significant wound area changes at days 3 and 4 (n = 10, p = 0.05, p = 0.025), respectively (Fig. 1C). In this study 48 h was chosen for detection of maximum skin pathology based on our recently published results from a mouse model of cutaneous nitrogen mustard exposure (Au et al., 2015). Histological evaluation of skin lesion 48 h following SM exposure at 50 mg/kg reveal death and erosion of the epidermal layer, disruption of hair follicles and inflammatory infiltration from SM mediated excessive tissue damage (Fig. 1D). Investigation of infiltrating cells in SM-inflamed skin sections by confocal microscopy showed a predominance of iNOS producing macrophages populating the dermis 48 h after exposure, suggesting an association between early tissue destruction and a predominance of iNOS± macrophages infiltrating the wound bed (Fig. 1E). This is supported by data from our recent publication demonstrating that cutaneous exposure to nitrogen mustard resulted in skin lesion that were exacerbated by activated macrophages in an iNOS-dependent manner (Au et al., 2015).

### 3.2. Systemic weight loss and bone marrow disruption following high dose SM exposure

**Fig. 2:**
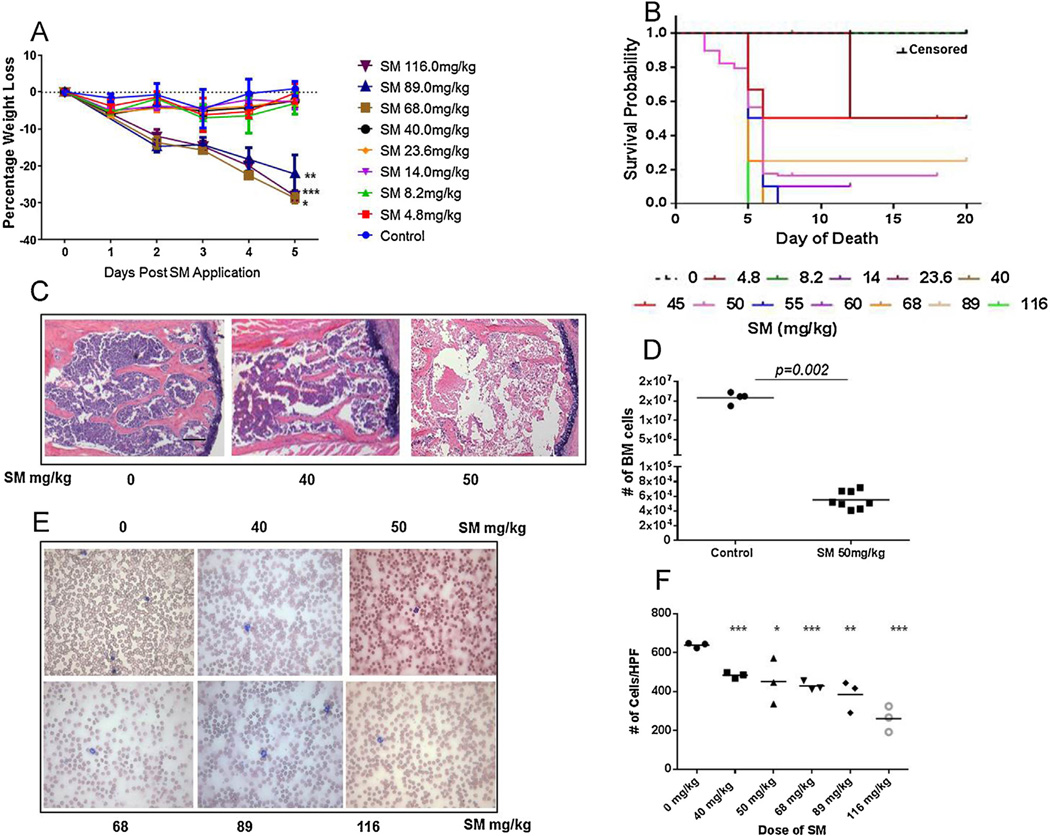
High dose SM exposure results in significant loss of body weight and increased mortality. A) Dose dependent loss in body weight *p = 0.039 for 89 mg/kg, **p = 0.009 for 68 mg/kg and ***p = 0.0002 for 116 mg/kg, compared to control, n = 4 per group, B) Dose dependent survival curve of mice treated with SM ranging from 4.8 mg/kg to 116 mg/kg, n = 10 for each group, p = 0.01 by Log Rank test. SM mediated systemic tissue destruction observed through C) loss of cellularity in H&E sections of bone marrow at 50 mg/kg SM dose 5 days post exposure, D) cell viability, n = 4 for controls, n = 8 for SM exposed animals, E) altered cellularity in peripheral blood of mice 5 days post SM exposure, F) graphical representation of cell number per HPF from blood smears,*p = 0.05, **p = 0.005, ***p ≤ 0.0006, compared to control, n = 3 per group. Scale bar 100 µm.

Mustard gas exposures have been associated with mortality consequently we investigated the systemic impact of SM exposure on skin. A daily progressive body weight loss of 4–6% and wasting was recorded in SM-exposed mice in a dose-dependent manner (Fig. 2A). Doses of 68 mg/kg and above corresponded with 100% mortality by day 5 (Kaplan Mieier curve, Fig. 2B), corroborating previous studies that demonstrated morbidity and body weight loss from high dose skin exposures to SM in mice (Goswami et al., 2015).

To establish a reproducible LD_90_ dose that would exert a dual effect of dermal destruction and systemic tissue destruction, mice were exposed to 4 doses of SM between 40 mg/kg (modest weight loss with no mortality) and 68 mg/kg (100% mortality by day 5) (data not shown). Interestingly, we observe no mortality with exposure to SM at doses ≤40 mg/kg, although doses ≥ 45 mg/kg appeared to be a critical threshold for death resulting in a stable LD_70_ at 50 mg/kg (Fig. 2B, pink line). Hematopoietic suppression in mustard gas victims has been the principal cause of SM associated mortality (Meisenberg et al., 1993) and our model recapitulates the phenotype such that cutaneous exposure to SM at 50 mg/kg induces acute loss of bone marrow cellularity as observed from H&E staining of sternum sections of SM mice 5 days post exposure (Fig. 2C). Parallel to sternal marrow disruption in SM mice, bone marrow cells isolated from the femur of SM exposed mice also exhibited acute cell depletion (analyzed by flow cytometry) (Fig. 2D). This observation was marked by cytopenia (Fig. 2E) with a 60% drop in peripheral red blood cells (261 cells per HPF in SM-exposed mice vs 640 cells per HPF in control mice, p < 0.005) (Fig. 2F). Finally, a 4-fold loss of white blood cells (WBC) and a 2-fold decrease of hematocrit (HCT) was recorded from CBC analysis supporting the evidence that skin exposure to SM precipitates in extensive and often fatal systemic pathology (Table 1).

### 3.3. Lesion area and weight loss can independently predict SM-induced mortality: protective role of 25(OH)D

**Fig. 3:**
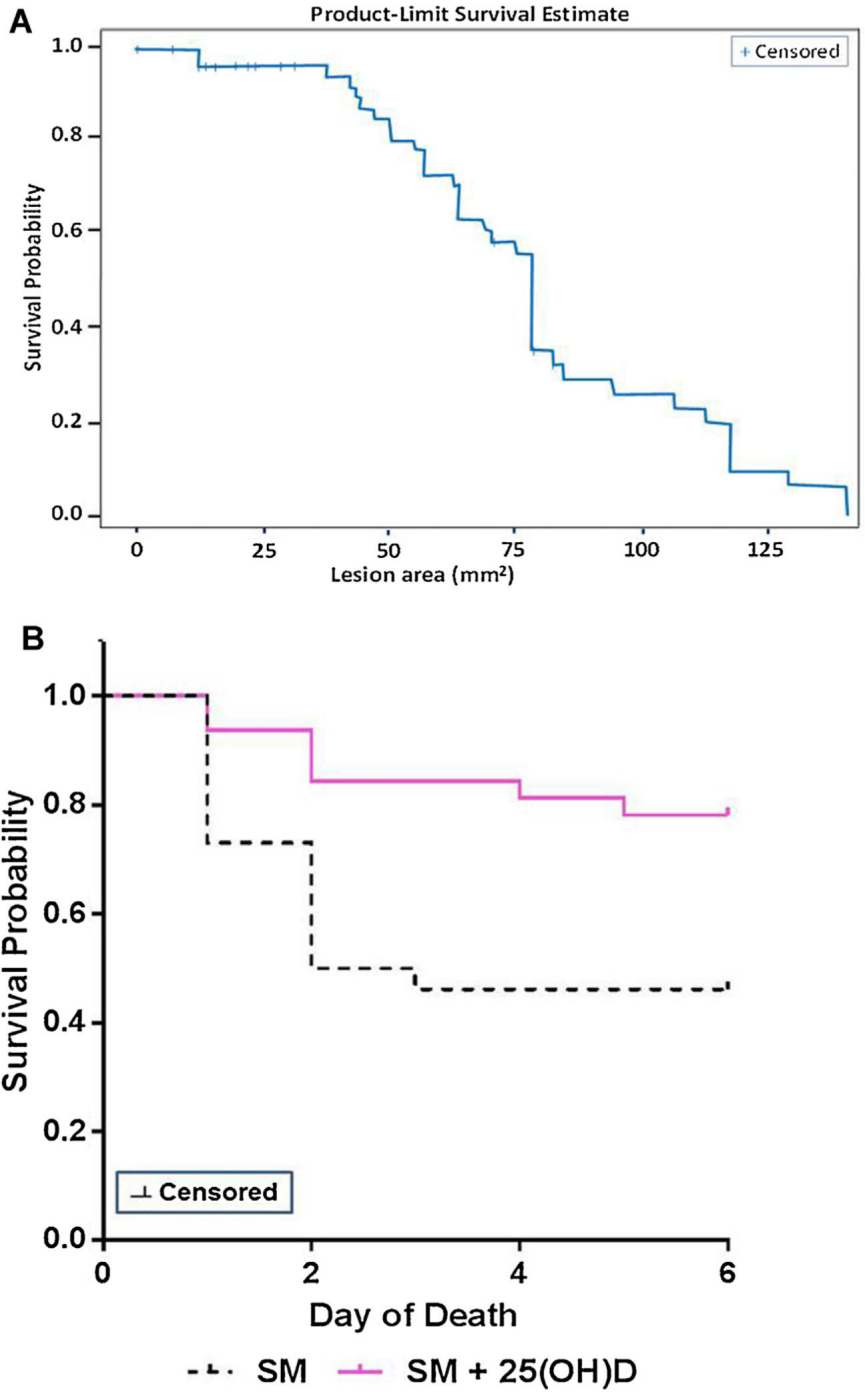
Lesion area as an independent predictor of mortality in presence and absence of a single dose of 25(OH)D. **A**) Cox regression analysis used lesion area at day 5 post exposure as an independent variable to develop a model to predict mortality of SM challenged mice at 50 mg/kg dose, n = 60. **B**) Intervention with 25(OH)D at 5 ng/ml 1 h post SM-exposure protects 40% of the mice from SM mediated death as assessed by a Log Rank test (p = 0.01), n = 26 for SM exposed animals, n = 32 for SM ± 25(OH)D treated animals.gr3

Based on progressive exacerbation of skin wound associated with increased wound area, the latter was considered an independent and statistically significant parameter to predict mortality following SM exposure (Fig. 3A). Using lesion size and maximum body weight loss as independent and statistically significant quantifiable outcomes, we developed a model to predict SM-induced lethality. The Cox regression analysis utilizes hazard ratios (HR) as a function of the variables (lesion size and body weight). HR <1 signifies inverse relationship between the predictor and its outcome whereas an HR >1 indicates a direct relationship. The HR for the weight variable (a surrogate for systemic pathology) was 0.391, indicating an expected inverse relationship between the weight and mortality. The HR for the lesion size (marker of local tissue destruction) was 1.017, indicative of a directly proportional relationship between the size of the lesion and mortality (Table 2).

The data presented defines 50 mg/kg SM in DMSO as the minimum dose that elicits a sufficient local (skin lesion) and systemic (blood & bone marrow alteration) response to generate an LD_70_ lethality model. Based on the efficacy of 25(OH)D in rescuing mice from NM-induced pathology (Au et al., 2015), 25(OH)D was used as potential countermeasure to mitigate the adverse effects of SM induced damage to skin and other organs. A single dose of 25 (OH)D at 5 ng/ml administered intraperitoneally (IP) demonstrated a moderate 40% protection from lethality that was statistically significant by (n = 60, Log Rank test, p = 0.01) (Fig. 3B). These data suggests that SM exposure imparts a skin pathology and weight loss that can be utilized in developing an LD_70_ model in mice that is attenuated by 25(OH)D.

## 4. Discussion

Our findings suggest that skin exposure to SM precipitates in an acute phenotype in mice characterized by skin ulceration, body weight loss and death, all of which progress in a dose dependent manner. We demonstrate that the early effects following challenge with SM include skin destruction that penetrates deep into the dermis through massive infiltration of activated macrophages producing elevated levels of iNOS. In most experimental animal models studied cutaneous wounds from SM burn progress with an inflammatory burst which critically influences the kinetics of wound healing (Mouret et al., 2014; Woessner et al., 1990). While models demonstrating vesicant-induced injury in lungs have been associated with inflammation induced iNOS production (Sunil et al., 2012), other studies suggest that iNOS production is inhibited by SM mediated skin injury (Ishida et al., 2008). Our NM skin exposure model established that iNOS plays a critical role in vesicant mediated skin tissue destruction (Au et al., 2015). Given the observation of skin infiltration with F4/80 + iNOS+ macrophages following SM exposure, we speculate that the immune system may play a role in exacerbating vesicant mediated injury. Erosion of the epidermal layer and inflammatory infiltration from SM mediated tissue damage are some of the classic features observed in studies on SKH-1 male mice exposed to SM vapor injury (Clery-Barraud et al., 2013). Although SM-mediated lung and ocular injuries have been recapitulated in several experimental animal models, percutaneous exposure to SM remains the principal and most rapid route of toxic penetration to inflict destruction to internal organs and blood (Langenberg et al., 1998). In accordance with other models of skin exposure to SM (Langenberg et al., 1998), our studies show that depletion of bone marrow cells correlate with cytopenia observed at day 5 post exposure. We establish from blood smear analyses that SM exposure depletes peripheral red blood cells by 60% (261 cells/HPF) compared to control mice (640 cells/HPF) (p < 0.005). The combination of the appearance of lesions, weight loss, cytopenia, bone marrow depression, and death were the parameters used for the acceptance of this model for use in the assessment of therapeutics against topical SM exposure. This study defined a dose of 50 mg/kg in DMSO to be an acceptable level for sufficient lesion formation and molecular insult to the bone marrow. Thus in this study we have established an optimal SM skin exposure model for tissue damage and death.

Vitamin D is widely available and has been shown to be beneficial in a NM skin vesication model and thus could have a broad public health impact in the event of chemical weapon exposure. A prospective study on patients with leg ulcers are associated with acute vitamin D deficiency that upon treatment with vitamin D shows a trend towards accelerated healing (Burkiewicz et al. 2012) suggesting that 25(OH)D may impact wound healing. Given the potential translational significance of vitamin D3 in suppressing skin and blood levels of iNOS and macrophages following exposure to nitrogen mustard, we developed a model to test whether 25(OH)D administered to SM-exposed mice may offer some protection against the deleterious effects of SM. The current work using one dose of 25(OH)D shows moderate protection from lethality. Ongoing work will determine if different dosing regimens can attenuate inflammation induced tissue destruction. One of the factors supporting enhanced inflammation in SM-exposed skin of Iranian war veterans is reportedly due to reduced expression levels of anti-inflammatory cytokines like TGF-β (Valizadeh et al., 2015) during the wound healing process. Our data contributes to that scenario by demonstrating that SM induced inflammation is associated with a surge of inflammatory infiltrates that may offset the action of the anti-inflammatory factors. Although medical advances have made attempts to counter the adverse effects of percutaneous exposure to mustard gas, treatment options to individuals are still dependent on late-appearing symptoms. To avoid situations of triaging patients based on severity of exposure, oral intake of a “safe” study drug like vitamin D by all victims would attenuate the first surge of inflammation and substantially reduce amplification of that response. In military zones with limited medical amenities, a rapid screening of patients that present with progressively increasing lesion sizes would be subject to further treatment compared to those with improved wound sizes. Vitamin D taken early on would potentially facilitate in containing the early immune responses and would be a key step in rescuing victims from the damaging effects of SM exposure.

In conclusion this study establishes that SM exposure to skin sustains skin vesication including formation of inflammatory foci at the site of exposure. Local skin destruction is accompanied by progressive weight loss that contributes to mortality. Our results provide the first evidence that early intervention with 25(OH)D offers protection from SM induced injury. Utilizing progressive increase in wound area and loss in body weight as the two key variables, our model presents a stable platform for advancing research to test the efficacy of potential countermeasures like 25(OH)D that would offer treatment options to exposed victims.

## Figures and Tables

**Table 1:** CBC analyses of SM exposed mouse on day 5 post exposure.

CBC analysis	Control	SM(50.0 mg/kg)
		
	n = 3	n = 4
WBC(×10^3^/µl)	3.21 ± 0.66	0.73 ± 0.16
HT(%)	27.60 ± 9.31	13.65 ± 3.87
Lymph (×10^3^/µl)	2.31 ± 0.54	0.42 ± 0.11
Mono (×10^3^/µl)	0.05 ± 0.02	0.01 ± 0.01

**Table 2:** Cox Regression Analysis. Body weight and lesion area are independent and statistically significant predictors of mortality.

Parameter	p-value	Hazard Ratio	95% Hazard Ratio Confidence Limits	
Weight at day 5	<0.0001	0.391	0.255	0.600
Lesion area at day 5	0.0489	1.017	1.000	1.035

## HIGHLIGHTS

- Skin wound area expansion and weight drop are predictors of death from sulfur mustard exposure and may have translational potential as non-invasive biomarkers.
- Skin exposure to sulfur mustard leads dose-dependent deleterious effects in the skin as well systemic toxicity including precipitous drop in body weight, peripheral blood cytopenia, and decreased bone marrow cellularity.
- Vitamin D is the first drug demonstrated to confer moderate protection from sulfur mustard-induced lethality and may have utility as a chemical countermeasure given its safety profile and current use in clinical practice.
